# Associations of prenatal per- and polyfluoroalkyl substances with whole blood folate levels in pregnant women in the Health Outcomes and Measures of the Environment (HOME) Study

**DOI:** 10.1097/EE9.0000000000000406

**Published:** 2025-06-30

**Authors:** Harin Lee, Amber M. Hall, Antonia M. Calafat, Aimin Chen, Zia Fazili, Bruce P. Lanphear, Christine M. Pfeiffer, Kimberly Yolton, Joseph M. Braun

**Affiliations:** a Hastings High School, Hastings-on-Hudson, New York, New York; b Department of Epidemiology, Brown University, Providence, Rhode Island; c National Center for Environmental Health, Centers for Disease Control and Prevention, Atlanta, Georgia; d Department of Biostatistics, Epidemiology and Informatics, Perelman School of Medicine, University of Pennsylvania, Philadelphia, Pennsylvania; e National Center for Environmental Health, Centers for Disease Control and Prevention, Atlanta, Georgia; f Faculty of Health Sciences, Simon Fraser University, Burnaby, British Columbia, Canada; g Department of Pediatrics, Cincinnati Children’s Hospital Medical Center, University of Cincinnati College of Medicine, Cincinnati, Ohio; h Department of Epidemiology, Brown University, Providence, Rhode Island

**Keywords:** Per- and polyfluoroalkyl substances, Folate, Pregnancy, Mixture analysis

## Abstract

**Background:**

Folate plays a critical role during pregnancy, preventing neural tube defects and possibly adverse neurodevelopment. Per- and polyfluoroalkyl substances (PFAS) are synthetic chemicals that may decrease folate levels. Although some studies have found associations between PFAS and folate, we are unaware of studies conducted in pregnant women. To address this knowledge gap, we evaluated associations between PFAS and whole blood folate (WBF) in pregnant women.

**Methods:**

We used data from 288 pregnant women in the Health Outcomes and Measures of the Environment (HOME) Study, a pregnancy and birth cohort in the Cincinnati Ohio area. We measured eight serum PFAS and WBF concentrations at 16 weeks’ gestation. We used linear regression to estimate the effect of each PFAS on WBF, and quantile-based g-computation and Bayesian kernel machine regression (BKMR) to investigate the joint effect of PFAS on WBF, adjusting for parity, prenatal vitamin intake, maternal race/ethnicity, household income, maternal age, and second trimester smoking status in all models. In addition, we investigated interactions between PFAS using BKMR.

**Results:**

We did not observe inverse associations of individual PFAS or their mixture with WBF, nor interactions between PFAS in the BKMR model in pregnant women.

**Conclusion:**

Future studies could consider WBF measures in late pregnancy to evaluate other periods of susceptibility. Furthermore, as people are exposed to multiple PFAS, future studies should continue to consider joint PFAS exposure.

**What this study adds**

Per- and polyfluoroalkyl substances (PFAS) are associated with several adverse health effects, including lower folate levels. However, no studies have investigated the impact of PFAS on folate levels during pregnancy, despite folate’s crucial role in the prevention of neural tube defects. We evaluated the impact of individual and joint serum PFAS on whole blood folate in pregnant women and did not find evidence of an inverse association. Strengths of our study include the evaluation of PFAS mixtures using two different methods, and the availability of multiple confounders, including prenatal vitamin intake, prenatal exposure to tobacco, and household income.

## Introduction

Per- and polyfluoroalkyl substances (PFAS) are synthetic chemicals that have been extensively manufactured since the 1940s for consumer and industrial applications.^1^ While the general population is mostly exposed to PFAS through food, exposure may also occur through contaminated drinking water, household dust, and consumer goods such as cosmetics and stain and water-resistant clothing.^2–6^ PFAS are ubiquitous, with around 97% of Americans having detectable serum PFAS concentrations.^7^ In addition, PFAS resist environmental degradation, and many are bioaccumulative with biological half-lives in humans that can range from 1.5 to 8.5 years.^8^ Therefore, understanding the impact of these ubiquitous, environmentally persistent, and often bioaccumulative chemicals on human health is crucial.

Exposure to PFAS has been linked to a wide range of human health effects at every life stage, with no known threshold of effect.^9–18^ In the general public, increasing PFAS levels have been associated with a greater risk of certain cancers, liver damage, immunotoxicity, and adverse cardiometabolic health.^10–14^ In pregnant women, higher PFAS concentrations have been associated with a greater risk of gestational hypertension and preeclampsia, gestational diabetes, altered gestational weight gain, and decreased birth weight.^10,15–18^ One study found a 1 ng/mL increase in perfluorooctanoic acid (PFOA) to be associated with 19 g (95% confidence interval [CI]: −29.8, −7.9) lower in birth weight.^19^ Maternal folate intake (vitamin B9) below the recommended adequate dietary intake of 600 µg/day has also been associated with reduced birth weight among other adverse pregnancy outcomes.^20^ Folate is essential to several biological processes including amino acid metabolism, DNA synthesis and repair, and cell division.^21,22^ Folate is especially important during pregnancy to support neurodevelopment and prevent neural tube defects (spina bifida and anencephaly) and red blood cell folate above 1000 nmol/L appears to be the threshold for minimizing neural tube defect risk.^23–26^ As both increasing PFAS levels and inadequate folate levels are associated with decreased birth weight, folate disruption could be a potential mechanism by which PFAS affects fetal growth and development. Moreover, prenatal PFAS exposure is of particular concern as chemical susceptibility to PFAS may be heightened during pregnancy compared with other life stages.^27^ Given the critical role of folate in necessary biological processes, including the development of the fetus’s neural tube, it is important to identify risk factors associated with folate dysregulation to protect pregnant women and their children. Understanding the impact of PFAS on pregnant women could lead to targeted recommendations and interventions.

Only three epidemiological studies have investigated the impact of PFAS on folate levels, with all studies observing significant associations between higher PFAS concentrations and lower folate levels for most of the PFAS evaluated.^28–30^ However, none of these studies were conducted in pregnant women, despite the critical role that folate plays during pregnancy.^28–30^ Furthermore, only one study has investigated the joint effects of PFAS (i.e., as a mixture) on folate levels, even though people are exposed to multiple PFAS simultaneously.^7,29^

To address these knowledge gaps, we investigated the associations of five serum PFAS concentrations with whole blood folate (WBF) levels at 16 weeks’ gestation in pregnant women in the Cincinnati, Ohio area, evaluating both the individual and joint (i.e., mixture analyses) effects of PFAS. As a secondary aim, we also investigated associations between two categorical PFAS concentrations with WBF. In addition, we explored the role of prenatal vitamin intake as well as maternal race/ethnicity as potential effect measure modifiers of the PFAS-folate relationship.

## Methods

### Study design

We used data from the Health Outcomes and Measures of the Environment (HOME) Study. The HOME Study is a pregnancy and birth cohort being conducted between 2003 and 2026.^31^ This study was designed to investigate the impact of environmental chemicals on children’s development and growth, and enrolled pregnant women in the greater Cincinnati, Ohio metropolitan area between March 2003 and February 2006. Eligibility for enrollment included living in a home built during or before 1978; being 16 ± 3 weeks pregnant; being ≥18 years old; planning on continuing care at one of nine prenatal practices; intending to live in the greater Cincinnati, Ohio metropolitan area for the next year; and speaking English fluently.^31^ Participants also had to be HIV-negative; off of medications for seizures or thyroid disorders; and have no diagnosis of diabetes, bipolar disorder, schizophrenia, or chemotherapy/radiation treatment-related cancer to be considered for inclusion.^31^ The mean week at which maternal venous blood samples were collected for both PFAS and folate assays was 16 weeks (range: 11–21 weeks). Before enrollment, the Cincinnati Children’s Hospital Medical Center’s Institutional Review Boards approved these research protocols. The Centers for Disease Control and Prevention (CDC) relied on Cincinnati Children’s Hospital Medical Center’s Institutional Review Boards, and pregnant women gave their written informed consent upon enrollment at each study visit. For this study, we included 288 women who met the inclusion criteria: having a WBF concentration, at least one measured PFAS concentration, and covariate information at 16 weeks’ gestation (Supplemental Figure 1 and Supplemental Table 1; https://links.lww.com/EE/A358).

### PFAS concentrations assessment

Procedures for quantifying serum PFAS concentrations have been previously published.^32–34^ In brief, maternal venous blood samples were collected from mothers during the time of recruitment at 16 weeks’ gestation.^31^ Serum was isolated from these samples and stored at −80 ºC, then shipped on dry ice to the CDC for testing.^32–35^ Serum concentrations of PFOA, perfluorononanoic acid (PFNA), perfluorodecanoic acid (PFDA), perfluorohexane sulfonic acid (PFHxS), perfluorooctane sulfonic acid (PFOS), perfluorooctanesulfonamide (FOSA), 2-(N-methyl-perfluorooctane sulfonamido) acetic acid (MeFOSAA), and 2-(N-ethyl-perfluorooctane sulfonamido) acetic acid (EtFOSAA) were measured using high-performance liquid chromatography-tandem mass spectrometry coupled with solid-phase extraction.^32^ All batches were analyzed along with analytical standards, quality control materials, and reagent blanks.^32^

The limit of detection (LOD) was 0.1 ng/mL for PFOA, PFNA, PFDA, PFHxS, FOSA, and EtFOSAA; 0.2 ng/mL for PFOS; and 0.09 ng/mL for MeFOSAA (Supplemental Table 2; https://links.lww.com/EE/A358). For this study, we included PFOS, PFOA, PFNA, PFHxS, and MeFOSAA in our primary analyses. For these PFAS, we imputed concentrations <LOD using the LOD/√2, and log_2_ transformed these values to reduce the influence of outliers. We excluded FOSA as <1% of samples had detectable concentrations (Supplemental Table 2; https://links.lww.com/EE/A358). Furthermore, we excluded EtFOSAA and PFDA in primary analyses as EtFOSAA had a low detection frequency (28.8%), and most PFDA concentrations were clustered around the value 0.2 ng/mL. Thus, the results from models of these PFAS could be biased if we treated them as continuous variables due to undue influence of values at a single concentration (Supplemental Table 2; https://links.lww.com/EE/A358). Although EtFOSAA and PFDA could also not be reliably analyzed continuously in single pollutant models, we conducted a secondary analysis with them as categorical variables; for these analyses, we categorized EtFOSAA concentrations as ≥LOD vs. <LOD and PFDA concentrations as <0.2, 0.2, and >0.2 ng/mL.

### Whole blood folate concentrations

We used high-performance liquid chromatography coupled with tandem mass spectrometry to quantify concentrations of five different folate vitamers in whole blood lysate samples.^36^ These folate vitamers included 5-methyltetrahydrofolate (5-methylTHF), folic acid, tetrahydrofolate (THF), 5-formyltetrahydrofolate (5-formylTHF), and 5,10-methenyltetrahydrofolate (5,10-methenylTHF).^37^ As 5-formylTHF coelutes with MeFox, we measured the sum of MeFox + 5-formylTHF.^37^ We summed these five vitamers and evaluated WBF concentrations as our outcome in the subsequently described statistical models.

The LODs (nmol/L; whole blood) of these folate forms were 5.2 for 5-methylTHF, 3.3 L for folic acid, 7.7 for THF, 13.3 for MeFox + 5-formylTHF, and 3.3 for 5,10-methenylTHF.^37^ We used the LOD/√2 to impute folate vitamer concentrations below the LOD.^37^

### Covariates

Trained staff administered standardized interviews to HOME Study participants during the 16 weeks’ gestation clinical visit to collect sociodemographic, health, and lifestyle information. This included data on prenatal vitamin intake (daily/less than daily), maternal race (white-non-Hispanic/other race), annual household income (USD), and maternal age (years). We also obtained data on parity (parous/nulliparous) through medical chart review. In addition, we measured serum cotinine concentrations, a biomarker for active and secondhand tobacco smoke exposure.^38^

We evaluated relations between exposure, outcome, and potential covariates using a directed acyclic graph (Supplemental Figure 2; https://links.lww.com/EE/A358).^39^ Based on this directed acyclic graph, we adjusted for parity, prenatal vitamin intake, maternal race, annual household income, maternal age, and tobacco smoke exposure (serum cotinine). For all analyses, serum cotinine concentrations were log_2_ transformed to reduce the influence of outliers.

### Statistical analyses

We calculated univariate statistics for all PFAS, as well as the frequency and percentage of detectable concentrations. We assessed correlations between log_2_-transformed concentrations of PFOS, PFOA, PFHxS, PFNA, and MeFOSAA using Pearson correlation coefficients. We also calculated the mean and standard deviation or frequency and percentage for WBF and all covariates, as appropriate. Furthermore, we calculated the mean WBF levels for each covariate category to investigate bivariate covariate-folate associations.

For our primary analyses, we estimated adjusted relations between individual serum PFAS and WBF using linear regression models. To determine the joint effect of exposure to multiple PFAS, we evaluated the association between the PFAS mixture (PFOA, PFOS, PFNA, PFHxS, and MeFOSAA) and WBF using quantile-based g-computation (QGcomp), adjusted for covariates.^40^ In addition, we calculated weights from this model to assess the individual contribution of each PFAS to the overall PFAS mixture. To examine consistency across different mixture models and evaluate interactions between individual PFAS, we used Bayesian kernel machine regression (BKMR), adjusting for covariates.^41^ We applied this model using a Gaussian kernel, Markov Chain Monte Carlo algorithm, and 50,000 iterations. We calculated posterior exclusion probabilities (PIPs) for each PFAS to investigate which PFAS (if any) meaningfully contributed to the overall PFAS mixture; PIPs >0.5 were considered meaningful contributors. We investigated the joint effect of the PFAS mixture by increasing all PFAS concentrations within the mixture simultaneously from the 25th to the 75th percentile. Next, we generated dose-response curves to better understand the relationship between each PFAS within the mixture and WBF, setting all other PFAS concentrations to their median. Finally, we explored potential interactions between PFAS by estimating the difference in WBF for a PFAS when another PFAS concentration was set at the 10th, 50th, and 90th percentiles, with the other three PFAS being set at their medians. We adjusted all models for parity, prenatal vitamin intake, maternal race, annual household income, maternal age, and smoking status.

As a secondary analysis, we evaluated adjusted relations between EtFOSAA and PFDEA, and WBF using linear regression models and adjusting for parity, prenatal vitamin intake, maternal race, annual household income, maternal age, and smoking status. We also explored potential effect measure modification (EMM) of PFAS-folate associations (on the additive scale) by prenatal vitamin intake and race/ethnicity. For prenatal vitamin intake, we aimed to ensure that differences between users and nonusers did not mask PFAS-folate associations and to determine if supplement use was a potential targeted intervention for PFAS-folate associations. Race/ethnicity might modify study results due to the history of systemic racism in the United States affecting various socioeconomic population characteristics. For this reason and to maintain consistency with previous studies, we also evaluated race/ethnicity as a modifier of the PFAS-folate association.^28,29^ We evaluated EMM by using interaction terms in our linear regression models, adjusting for the same covariates. For this assessment, an *a priori P*-interaction <0.10 was considered statistically significant. All statistical analyses were conducted using R version 4.3.0.^42^

## Results

**Figure 1.**
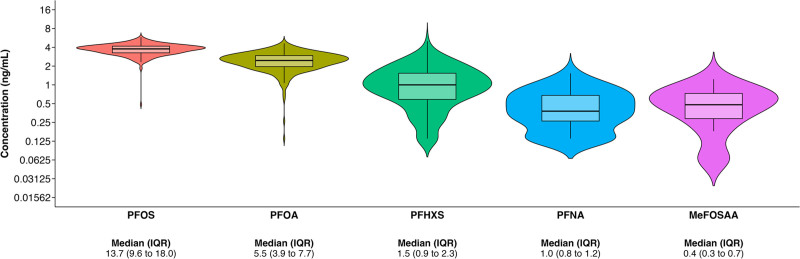
Serum PFAS concentrations (ng/mL) in pregnant women at 16 weeks’ gestation in the HOME Study (2003–2006), N = 288. HOME indicates Health Outcomes and Measures of the Environment Study; IQR, interquartile range; MeFOSAA, 2-(N-Methyl-perfluorooctane sulfonamido) acetic acid; PFAS, per- and polyfluoroalkyl substances; PFHxS, perfluorohexane sulfonic acid; PFNA, perfluorononanoic acid; PFOA, perfluorooctanoic acid; PFOS, perfluorooctane sulfonic acid.

Median serum concentrations were highest for PFOA (5.5 ng/mL) and lowest for FOSA (<LOD) (Figure 1 and Supplemental Table 2; https://links.lww.com/EE/A358). All five primary PFAS (PFOS, PFOA, PFHxS, PFNA, and MeFOSAA) had detectable concentrations in >99% of samples (Supplemental Table 2; https://links.lww.com/EE/A358). Pearson correlation coefficients for these PFAS ranged from *r* = 0.16 (MeFOSAA and PFOA) to *r* = 0.63 (for PFHxS and PFOS) (Supplemental Figure 3; https://links.lww.com/EE/A358).

The average age for study participants was 29 years and most women were parous (55.6%), non-Hispanic white (59.4%), had an annual household income of more than $40,000 (61.1%), used prenatal vitamins daily (71.2%), and did not smoke (91.7%) (Table 1). The average WBF concentration for participants was 451 nmol/L (SD: 224.6), and WBF levels were consistently lower for participants who did not identify as non-Hispanic white, had a lower annual household income, smoked during pregnancy, were parous, and did not take prenatal vitamins daily (Table 1).

**Table 1.** Sociodemographic characteristics and whole blood folate (WBF) levels for HOME Study participants at 16 weeks’ gestation (2003–2006), N = 288

	N (%)	WBF (nmol/L) mean (SD)
Maternal age at birth (years)		
<30	149 (51.70)	407.5 (206.2)
30–34	79 (27.40)	488.7 (231.7)
>34	60 (20.8)	507.0 (240.5)
Maternal race/ethnicity		
White, non-Hispanic	171 (59.4)	508.7 (245.1)
Another race/ethnicity	117 (40.6)	365.5 (156.5)
Annual household income (USD)		
<20,000	61 (21.2)	371.1 (150.9)
20,000–40,000	51 (17.7)	409.9 (230.3)
40,000–80,000	98 (34.0)	462.1 (228.7)
>80,000	78 (27.1)	524.7 (241.0)
Gestational tobacco exposure (cotinine [ng/mL])		
<0.015 (unexposed)	76 (26.4)	504.9 (243.5)
0.015–3 (secondhand)	188 (65.3)	445.3 (219.6)
>3 (active smoker)	24 (8.3)	319.9 (127.3)
Prenatal vitamin use		
<Daily	83 (28.8)	377.5 (164.5)
Daily	205 (71.2)	480.1 (238.8)
Parity		
Nulliparous	128 (44.4)	469.8 (228.8)
Parous	160 (55.6)	435.1 (220.7)

HOME, Health Outcomes and Measures of the Environment Study; N, number; SD, standard deviation; USD, United States Dollar.

**Figure 2.**
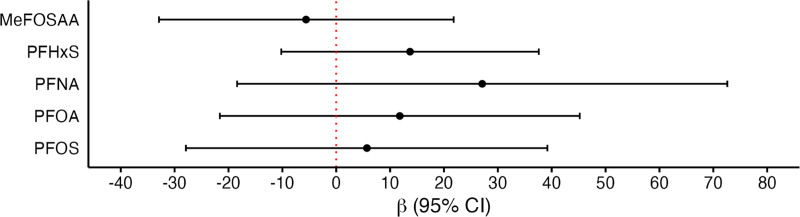
Adjusted difference in WBF (nmol/L) with higher serum PFAS concentrations among pregnant women (16 weeks’ gestation) in the HOME Study (2003–2006), N = 288. PFAS concentrations are log2-transformed and concentrations below the LOD are imputed with LOD/√2. All models are linear regression models, adjusted for parity, prenatal vitamin intake, maternal race, annual household income, maternal age, and smoking status in this analysis. EtFOSAA indicates 2-(N-ethyl-perfluorooctane sulfonamido) acetic acid; HOME, Health Outcomes and Measures of the Environment Study; MeFOSAA, 2-(N-methyl-perfluorooctane sulfonamido) acetic acid; PFAS, per- and polyfluoroalkyl substances; PFDA, perfluorodecanoic acid; PFHxS, perfluorohexane sulfonic acid; PFNA, perfluorononanoic acid; PFOA, perfluorooctanoic acid; PFOS, perfluorooctane sulfonic acid; WBF, whole blood folate.

In our analyses of individual PFAS and WBF, we observed null associations for all five PFAS (Figure 2 and Supplemental Table 2; https://links.lww.com/EE/A358). Using QGcomp, we did not find any associations between the PFAS mixture (PFOA, PFOS, PFNA, PFHxS, and MeFOSAA) and WBF (Supplemental Figure 4; https://links.lww.com/EE/A358).

**Figure 3.**
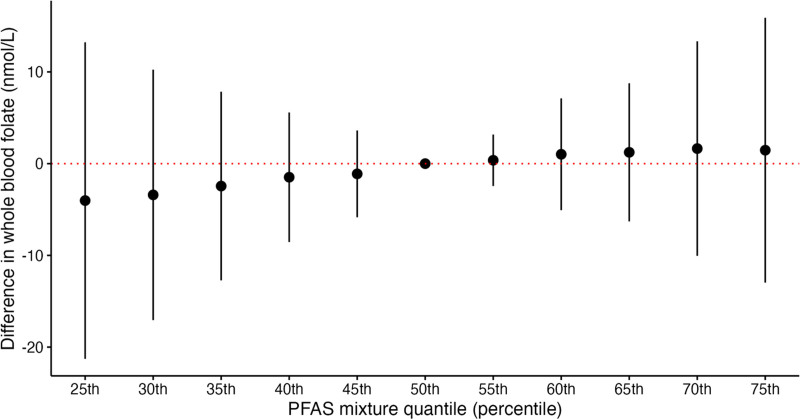
Adjusted differences in WBF levels (nmol/L) by PFAS mixture concentration quantile (25th–75th percentile) among pregnant women (16 weeks’ gestation) in the HOME Study (2003–2006) using BKMR, N = 288. The BKMR model evaluated a mixture of 5 PFAS (PFOS, PFOA, PFNA, PFHxS, and MeFOSAA) on whole blood folate levels, adjusted for parity, prenatal vitamin intake, maternal race/ethnicity, household income, maternal age, and log 2 maternal cotinine. The model used a Gaussian kernel, Markov Chain Monte Carlo algorithm, and ran 50,000 iterations. BKMR indicates Bayesian kernel machine regression; HOME, Health Outcomes and Measures of the Environment Study; PFAS, per- and polyfluoroalkyl substances; WBF, whole blood folate.

**Figure 4.**
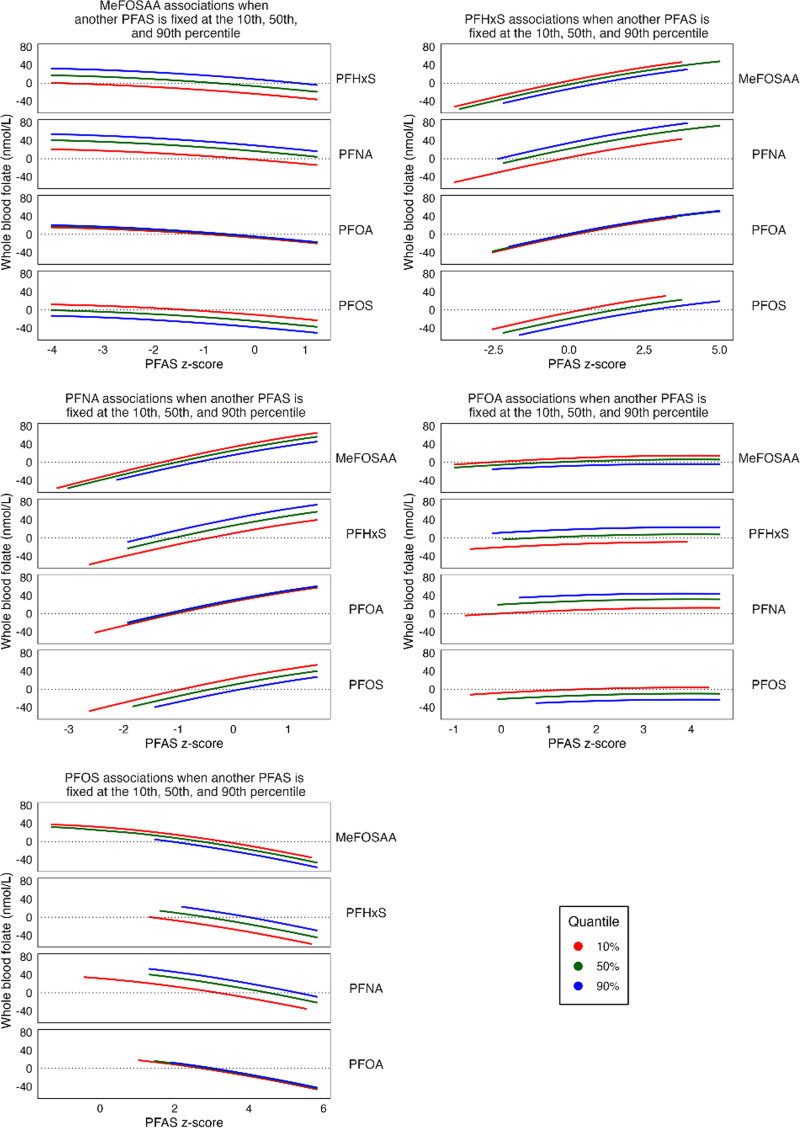
Adjusted associations of each PFAS serum concentrations with WBF levels (nmol/L), with other PFAS concentrations fixed at the 10th, 50th, and 90th percentiles, in pregnant women (16 weeks’ gestation) in the HOME Study (2003–2006) using BKMR, N = 288. The BKMR model evaluated a mixture of 5 PFAS (PFOS, PFOA, PFNA, PFHxS, and MeFOSAA) on whole blood folate levels, adjusted for parity, prenatal vitamin intake, maternal race/ethnicity, household income, maternal age, and log 2 maternal cotinine. The model used a Gaussian kernel, Markov Chain Monte Carlo algorithm, and ran 50,000 iterations. BKMR indicates Bayesian kernel machine regression; HOME, Health Outcomes and Measures of the Environment Study; MeFOSAA, 2-(N-methyl-perfluorooctane sulfonamido) acetic acid; PFAS, per- and polyfluoroalkyl substances; PFHxS, perfluorohexane sulfonic acid; PFNA, perfluorononanoic acid; PFOA, perfluorooctanoic acid; PFOS, perfluorooctane sulfonic acid; WBF, whole blood folate.

Consistent with the QGcomp model, we did not observe any significant associations for joint PFAS effects or individual PFAS effects and WBF levels using BKMR (Figures 3 and 4 and Supplemental Figure 5, https://links.lww.com/EE/A358). Furthermore, no individual PFAS meaningfully contributed to the overall PFAS mixture, with the highest PIP observed for PFNA (0.25), and all other PIPs between 0.13 and 0.14 (Supplemental Table 4; https://links.lww.com/EE/A358). We did not observe any interactions between PFAS in this model of the PFAS-folate relationship.

In our secondary analyses, we observed a positive association with no evidence of a dose-response relationship for prenatal PFDA concentrations with WBF (<0.2 ng/mL: β = 67.4 [95% CI: 4.5, 130.4]; >2.0 ng/mL: β = 51.4 [95% CI: −18.4, 120.3]) (Supplemental Table 3; https://links.lww.com/EE/A358). We observed a null association for EtFOSAA (Supplemental Table 3; https://links.lww.com/EE/A358), and did not observe any EMM of the PFAS-folate relationship by race/ethnicity or prenatal vitamin intake (Supplemental Tables 5 and 6; https://links.lww.com/EE/A358).

## Discussion

We did not observe evidence of inverse associations between PFAS and WBF in these second trimester pregnant women from the Cincinnati, Ohio area. While higher serum PFDA concentrations were associated with higher WBF levels, but we did not observe evidence of a dose-response relationship and the range of PFDA concentrations was narrower than that for other PFAS. Furthermore, we did not find evidence of EMM by prenatal vitamin intake nor race and ethnicity for the PFAS-WBF relationship. We also did not observe a significant association between the PFAS mixture and WBF using either QGcomp or BKMR.

As PFAS are ubiquitous in the environment and pose various health risks for the mother and fetus, it is important to study the interactions between PFAS and essential biological functions. Previous studies on PFAS and folate observed inverse associations using cross-sectional data from the National Health and Nutrition Examination Survey (NHANES) (Supplemental Table 7; https://links.lww.com/EE/A358).^28–30^ Jain^28^ evaluated the relationship between serum PFAS concentrations and red blood count (RBC) folate levels in U.S. adults aged ≥20 years between 2007 and 2014, finding inverse associations of PFOA, PFOS, PFDA, PFHxS, and PFNA with RBC folate. Similarly, Tian et al^29^ observed inverse associations of PFOS and PFDA serum concentrations and a PFAS mixture (PFHxS, PFOS, PFOA, PFNA, and PFDA) with RBC folate, as well as inverse associations between PFDA and serum folate in adolescents aged 12–19 years from 2007 to 2010. Finally, Zhang et al^30^ observed inverse associations between PFOA, PFOS, and PFNA serum concentrations with both serum folate and RBC folate, as well as PFHxS with RBC folate in adults from 2003 to 2016. Similarly, in the same study, Zhang et al^30^ observed inverse associations between PFOS with serum folate and RBC folate, as well as inverse associations between PFNA and RBC folate.

Our study’s results differ from previous studies as we did not observe inverse associations of serum PFAS with WBF. In a secondary analysis, our study observed a positive association between PFDA concentrations and WBF levels, but without evidence of a dose-response relationship. This finding was unexpected and inconsistent with findings by Jain^28^ and Tian et al^29^ that observed inverse associations of PFDA with RBC folate and serum folate (PFDA was not evaluated by Zhang et al). Although Tian et al^29^ observed an inverse association of serum PFDA with serum and RBC folate, this association was observed when PFDA was evaluated continuously, not categorically (Tian et al categorized PFDA as low [<LOD to 0.2 ng/mL], medium [0.2–0.3 ng/mL], and high [0.3–2.3 ng/mL]). As such, analyzing PFDA as a categorical variable may have introduced misclassification bias in our study, leading to unexpected results. However, as 125 of our participants (43%) had a PFDA concentration of 0.2 ng/mL and 84% had PFDA concentrations of 0.1, 0.2, or 0.3 ng/mL, we did not feel analyzing PFDA continuously was appropriate. Thus, studies with a greater degree of precision in PFDA measurement or a wider range of PFDA concentrations might be better suited to evaluate the impact of PFDA on folate. In addition, it should be noted that Jain^28^ and Zhang et al^30^ excluded pregnant women from their study, and Tian et al^29^ evaluated associations in adolescents, a population that may differ from adults and/or pregnant women. We speculate that the lack of associations in our study may, in part, be due to pregnant women having greater folate intake than nonpregnant women and higher rates of supplement use that increase across the course of pregnancy.^20,43,44^ Thus, pregnant women may be less susceptible to PFAS-related changes in folate status. Therefore, differences between these studies being the result of population-level differences cannot be dismissed.

The results from our study were similar to Tian et al,^29^ as neither our study nor theirs observed an association between joint PFAS concentrations and whole blood or serum folate concentrations using two mixture methods (QGcomp and BKMR). However, Tian et al did observe an association for joint PFAS concentrations and RBC folate. As our study’s WBF measure predominantly reflects RBC folate, which is more stable over time, this observed difference was unexpected. However, differences between our results and Tian’s may be due to sample size as Tian’s study has 721 participants and our study had 288; thus, Tian’s study was powered to detect smaller differences compared with our study. Therefore, future studies with larger sample sizes would benefit from evaluating associations between PFAS and folate in pregnant women. Moreover, it should be noted that the joint effects between our mixture and Tian’s mixture are not directly comparable, as Tian et al^29^ included PFDA in addition to four other PFAS (PFOS, PFOA, PFNA, and PFHxS), and we included MeFOSAA in addition to these four PFAS. However, as no other studies have evaluated associations between PFAS as a mixture and folate, no other study was available for comparative purposes. Furthermore, Tian’s study noted significant interactions between PFAS when evaluating PFAS-folate associations, while our study observed no interactions between PFAS.^29^ This difference between our study and Tian’s may be due to population-level differences in PFAS exposures between the Cincinnati, Ohio area and the USA in general. For instance, participants in the HOME study experienced higher exposure to PFOA from contaminated drinking water than the general U.S. population.^45^ However, given Tian et al’s findings and the ubiquity of PFAS in the population, it is useful for future studies to consider potential interactions between PFAS with relation to the PFAS-folate relationship.^29^

Previous research on modification of the PFAS-folate association by race and/or ethnicity using NHANES data has been inconclusive.^28,29^ Jain^28^ observed significant modification, with persons identifying as non-Hispanic Black having stronger inverse PFAS-folate associations compared with those identifying as other races. However, neither our study nor Tian’s study observed modification by race or ethnicity.^29^ Differences between Jain’s, Tian et al’s, and our studies may be the results of how we evaluated modification. For instance, Jain^28^ did not conduct formal testing between groups for differences in PFAS-folate associations. Therefore, conclusions from this study were drawn from visual assessment, which may be less precise.^28^ Furthermore, it should also be noted that our study had a substantially smaller sample size (N = 288) compared with Jain’s (N = 6291) and Tian’s (N = 721), which both used data from NHANES.^28,29^ Thus, the null results in our study may be the result of being underpowered to detect smaller PFAS-folate differences compared with Jain’s and Tian et al’s studies. In addition to race/ethnicity, our study was the first study to investigate the modification of PFAS-folate associations by prenatal vitamin intake, finding no associations. However, Zhang et al^30^ did note that their observations appeared to be stronger for those taking a folic acid supplement compared with those who did not. Therefore, future studies should consider investigating PFAS-folate associations by race and ethnicity as well as supplementation such as taking a folate supplement or prenatal vitamin.

There are multiple strengths to this study. First, we adjusted for multiple confounders including parity, prenatal vitamin intake, race, annual household income, age, and tobacco smoke exposure, reducing confounding bias in our statistical models. In addition, we investigated the potential additive EMM by parity and maternal race/ethnicity, providing insight into whether PFAS-folate associations were modified by these covariates. We also evaluated multiple PFAS, allowing us to better characterize the potential effects of PFAS on folate. In addition, we evaluated joint effects from a PFAS mixture on folate levels using two commonly used mixture methods. As people are exposed to multiple PFAS, analyzing PFAS mixtures is more reflective of everyday exposure.^46^ Furthermore, the use of two mixture methods allowed us to validate our findings across models. Finally, we were able to investigate potential interactions between PFAS in our assessment of the PFAS mixture on WBF, allowing us to identify any interactions that occurred between these PFAS in relation to the PFAS-WBF association.

This study has certain limitations that should be acknowledged. We conducted this study with a modest sample size compared with previous studies.^28–30^ As such, this could have limited our ability to detect subtle PFAS-folate associations. Another limitation is that our analyses were cross-sectional, limiting causal inference. However, we are unaware of other studies assessing the impact of PFAS on folate levels in pregnant women, thus, this study contributes valuable information regardless of this limitation. In addition, we conducted our study only in the Cincinnati Ohio area, so findings from this study may not be generalizable to other populations. However, most serum PFAS concentrations were generally consistent with those observed in the US population at the same calendar time.^47^ Furthermore, adverse association between PFAS and some neonatal outcomes (e.g., fetal growth) may be confounded by maternal renal function (measured via glomerular filtration rate) and we were unable to adjust for this.^48^ However, this is unlikely to have impacted our results as most of our associations were null. We were also likely underpowered to detect modification of the PFAS-folate relationship by race and ethnicity as well as prenatal vitamin intake. Therefore, care should be taken in the interpretation of the null modification results in this study.

In conclusion, we did not observe that individual serum PFAS concentrations or their mixture were inversely associated with WBF levels in this study of pregnant women. Furthermore, we did not observe evidence of modification of this relationship by prenatal vitamin intake or race/ethnicity. Future studies could consider multiple folate measures or WBF measures in different time periods of pregnancy to evaluate heightened periods of susceptibility. In addition, future studies could be conducted in folate-deficient populations which might be more susceptible.

## Conflicts of interest statement

J.M.B. received compensation as an expert witness for plaintiffs involved in litigation regarding PFAS-contaminated drinking water. No other authors have competing interests to declare. The remaining authors declare that they have no conflicts of interest with regard to the content of this report.

## Supplementary Material
