# Supplementary material for: Associations of prenatal per- and polyfluoroalkyl substances with whole blood folate levels in pregnant women in the Health Outcomes and Measures of the Environment (HOME) Study
Source: Environ Epidemiol. 2025 Jun 30;9(4):e406. doi: 10.1097/EE9.0000000000000406 (PMC12212838; doi:10.1097/EE9.0000000000000406)
Supplement: Supplementary file 1 [file ee9-9-e406-s001.pdf]

Supplemental Material For: Associations of prenatal per- and polyfluoroalkyl substances with whole blood folate levels in pregnant women in the Health Outcomes and Measures of the Environment (HOME) Study

Authors: Harin Lee, Amber M. Hall, Antonia M. Calafat, Aimin Chen, Zia Fazili, Bruce P. Lanphear, Christine M. Pfeiffer, Kimberly Yolton, and Joseph M. Braun

Supplemental Table 1. Participant characteristics stratified by inclusion in analytic sample.

	Included (n=288)	Excluded (n=51)	Total* (n=339)
Maternal age at birth (years)			
Mean (sd)	29.3 (5.8)	28.4 (5.6)	29.2 (5.8)
Maternal race/ethnicity			
White, non-Hispanic	171 (59.4)	29 (56.9)	200 (59)
Another race/ethnicity	117 (40.6)	22 (43.1)	139 (41)
Annual household income (USD)			
Median [iqr]	55,000 [62,500]	45,000 [57,500]	55,000 [62,500]
Parity			
Nulliparous	128 (44.4)	20 (39.2)	148 (43.7)
Parous	160 (55.6)	29 (56.9)	189 (55.8)
Missing	0	2	2
Gestational tobacco exposure [cotinine (ng/mL)]			
Median [iqr]	0.03 [0.21]	0.09 [47.5]	0.04 [0.3]
Missing	0	8	8
Prenatal vitamin use			
<Daily	83 (28.8)	17 (33.3)	100 (29.5)
Daily	205 (71.2)	34 (66.7)	239 (70.5)
WBF (nmol/L)			
Mean [sd]	450.6 [224.6]	483 [212.9]	455.4 [222.9]
EtFOSAA (ng/L)			
Median [iqr]	0.1 [0.1]	0.1 [0.1]	0.1 [0.1]
Missing	205	40	245
MeFOSSA (ng/L)			
Median [iqr]	0.4 [0.4]	0.6 [0.5]	0.4 [0.4]
Missing	0	3	3
PFDA (ng/L)			
Median [iqr]	0.2 [0.1]	0.2 [0]	0.2 [0.1]
Missing	13	13	26
PFHxS (ng/L)			
Median [iqr]	1.5 [1.4]	1.2 [1]	1.5 [1.4]
Missing	0	3	3
PFNA (ng/L)			
Median [iqr]	1 [0.4]	0.8 [0.4]	0.9 [0.5]
Missing	0	3	3
PFOA (ng/L)			
Median [iqr]	5.5 [3.8]	5 [3]	5.5 [3.9]
Missing	0	3	3
PFOS (ng/L)			
Median [iqr]	13.7 [8.4]	11.05 [10]	13.5 [8.8]
Missing	0	3	3
FOSA (ng/L)			
Median [iqr]	0.2 [0]	NA	0.2 [0]
Missing	287	51	338

Supplemental Table 2. Serum PFAS concentrations (ng/mL) in pregnant women at 16 weeks' gestation in the HOME Study (2003-2006), N=288

PFAS	Min	25th	50th	75th	Max	LOD	N (%) ≥ LOD
PFOA	0.5	3.9	5.5	7.7	25	0.1	288 (100.0%)
PFNA	0.1	0.8	1.0	1.2	2.9	0.1	288 (100.0%)
PFDA	<LOD	0.2	0.2	0.3	1.3	0.1	275 (95.5%)
PFHxS	<LOD	0.9	1.5	2.3	33	0.1	287 (99.7%)
PFOS	0.4	9.6	14	18	57	0.2	288 (100.0%)
FOSA	<LOD	<LOD	<LOD	<LOD	0.2	0.1	1 (0.3%)
MeFOSAA	<LOD	0.3	0.4	0.7	2.4	0.09	287 (99.7%)
EtFOSAA	<LOD	<LOD	<LOD	0.2	2.2	0.1	83 (28.8%)

Note: PFAS, per- and polyfluoroalkyl substances; ng/mL, nanograms per milliliter; HOME, Health Outcomes and Measures of the Environment Study; Min, minimum; Max, maximum; LOD, limit of detection; N, number; PFOA, perfluorooctanoic acid; PFNA, perfluorononanoic acid; PFDA, perfluorodecanoic acid; PFHxS, perfluorohexane sulfonic acid; PFOS, perfluorooctane sulfonic acid; FOSA, perfluorooctanesulfonamide; MeFOSAA, 2-(N-Methyl-perfluorooctane sulfonamido) acetic acid; EtFOSAA, 2-(N-Ethyl-perfluorooctane sulfonamido) acetic acid.

Supplemental Table 3. Adjusted differences in WBF levels (nmol/L) with higher serum PFAS levels at 16 weeks' gestation, HOME Study (2003-2006), N=288

Log2- PFAS	*Crude Effect Estimate (95% CI)*	*Adjusted Effect Estimate (95% CI)*
**PFOA**	24.1 (-9.3 to 57.5)	11.8 (-21.6, 45.2)
**PFOS**	28.9 (-4.9 to 62.7)	5.7 (-27.9 to 39.2)
**PFNA**	54.1 (8.1 to 100.2)	27.1 (-18.4 to 72.6)
**PFHxS**	31.9 (8.5 to 55.3)	13.7 (-10.2 to 37.6)
**MeFOSAA**	-12.6 (-41.4 to 16.2)	-5.6 (-32.9 to 21.8)
Categorical PFAS		
**PFDA**		
<0.2ng/mL	REF	REF
0.2ng/mL	103.9 (39.9, 167.9)	67.4 (4.5 to 130.4)
>0.2ng/mL	103.8 (35.3 to 172.2)	51.0 (-18.4 to 120.3)
**EtFOSAA**		
<LOD	REF	REF
≥ LOD	-21.5 (-79.1 to 36.1)	-32.2 (-87.3 to 22.9)

Note: WBF, whole blood folate; PFAS, per- and polyfluoroalkyl substances; HOME, Health Outcomes and Measures of the Environment Study; REF, reference; CI, confidence interval; ng/mL, nanograms per milliliter; PFOS, perfluorooctane sulfonic acid; PFOA, perfluorooctanoic acid; PFNA, perfluorononanoic acid; PFHxS, perfluorohexane sulfonic acid; MeFOSAA, 2-(N-Methyl-perfluorooctane sulfonamido) acetic acid; PFDA, perfluorodecanoic acid; EtFOSAA, 2-(N-Ethyl-perfluorooctane sulfonamido) acetic acid.

PFAS were log-2 transformed, except for PFDA and EtFOSAA, which were analyzed as categorical variables.

Adjusted models were adjusted for parity, prenatal vitamin intake, maternal race/ethnicity, household income, maternal age, and log 2 maternal cotinine.

Supplemental Table 4. PIPs of each PFAS in the BKMR model in the HOME Study (2003-2006), N=288

PFOS	0.14
PFOA	0.13
PFNA	0.25
PFHxS	0.13
MeFOSAA	0.13

Note. PIP, posterior inclusion probability; PFAS, per- and polyfluoroalkyl substances; BKMR, Bayesian Kernel Machine Regression; HOME, Health Outcomes and Measures of the Environment; PFOS, perfluorooctane sulfonic acid; PFOA, perfluorooctanoic acid; PFNA, perfluorononanoic acid; PFHxS, perfluorohexane sulfonic acid; MeFOSAA, 2-(N-Methyl-perfluorooctane sulfonamido) acetic acid.

The BKMR model evaluated a mixture of 5 PFAS (PFOS, PFOA, PFNA, PFHxS, MeFOSAA) on total folate levels, adjusted for parity, prenatal vitamin intake, maternal race/ethnicity, household income, maternal age, and log 2 maternal cotinine.

The model used a Gaussian kernel, Markov Chain Monte Carlo algorithm, and ran 50,000 iterations.

Supplemental Table 5. Adjusted differences in WBF levels (nmol/L) with higher serum PFAS levels at 16 weeks' gestation, HOME Study (2003-2006), stratified by maternal race

Log2- PFAS	Adjusted Effect Estimate (95% CI)		P-interaction
	White, Non-Hispanic (N=171)	*Other (N=117)*	
**PFOA**	26.3 (-24.2, 76.8)	1.1 (-42.6, 44.7)	0.45
**PFOS**	13.6 (-31.1, 58.4)	-4.2 (-53.7, 45.4)	0.60
**PFNA**	35.2 (-27.2, 97.6)	18.2 (-47.2, 83.6)	0.71
**PFHxS**	33.8 (-0.7, 68.3)	-4.1 (-36.6, 28.3)	0.11
**MeFOSAA**	6.7 (-38, 51.3)	-13.0 (-47.7, 21.8)	0.50
Categorical PFAS			
**PFDA**			
<0.2ng/mL	REF	REF	
0.2ng/mL	49.7 (-40.7, 140.1)	86.0 (-3.0, 175.1)	0.57
>0.2ng/mL	31.6 (-74.6, 137.8)	68.9 (-23.6, 161.5)	0.60
**EtFOSAA**			
<LOD	REF	REF	
≥ LOD	-7.6 (-97.5, 82.2)	-47.0 (-116.9, 22.9)	0.50

Note: WBF, whole blood folate levels; PFAS, per- and polyfluoroalkyl substances; HOME, Health Outcomes and Measures of the Environment Study; REF, reference; CI, confidence interval; ng/mL, nanograms per milliliter; PFOS, perfluorooctane sulfonic acid; PFOA, perfluorooctanoic acid; PFNA, perfluorononanoic acid; PFHxS, perfluorohexane sulfonic acid; MeFOSAA, 2-(N-Methyl-perfluorooctane sulfonamido) acetic acid; PFDA, perfluorodecanoic acid; EtFOSAA, 2-(N-Ethyl-perfluorooctane sulfonamido) acetic acid.
PFAS were log-2 transformed, except for PFDA and EtFOSAA, which were analyzed as categorical variables.
Adjusted models were adjusted for parity, prenatal vitamin intake, maternal race/ethnicity, household income, maternal age, log 2 maternal cotinine, and an interaction term for the PFAS of interest and prenatal vitamin intake.
A p-interaction <0.10 was considered statistically significant.

Supplemental Table 6. Adjusted differences in WBF levels (nmol/L) with higher serum PFAS levels at 16 weeks' gestation, HOME Study (2003-2006), stratified by prenatal vitamin intake

Log2- PFAS	Adjusted Effect Estimate (95% CI)		P-interaction
	Less than daily (N=83)	*Daily (N=205)*	
**PFOA**	8.0 (-30.5, 46.6)	21.9 (-39.3, 83.2)	0.70
**PFOS**	3.8 (-37.8, 45.4)	8.9 (-45.8, 63.6)	0.88
**PFNA**	16.0 (-40.2, 72.2)	46.9 (-27.5, 121.4)	0.51
**PFHxS**	7.0 (-21.0, 34.9)	29.8 (-12.3, 71.8)	0.36
**MeFOSAA**	-9.3 (-41.2, 22.6)	5.0 (-48.5, 58.4)	0.65
Categorical PFAS			
**PFDA**			
<0.2ng/mL	REF	REF	
0.2ng/mL	66.8 (-9.0, 142.5)	66.8 (-43.8, 177.4)	1.00
>0.2ng/mL	40.8 (-40.6, 122.2)	79.1 (-46.2, 204.3)	0.61
**EtFOSAA**			
<LOD	REF	REF	
≥ LOD	-42.3 (-107.9, 23.2)	-7.8 (-109.1, 93.4)	0.57

Note: WBF, whole blood folate; nmol/L, nanomoles per liter; PFAS, per- and polyfluoroalkyl substances; HOME, Health Outcomes and Measures of the Environment Study; REF, reference; CI, confidence interval; PFOS, perfluorooctane sulfonic acid; PFOA, perfluorooctanoic acid; PFNA, perfluorononanoic acid; PFHxS, perfluorohexane sulfonic acid; MeFOSAA, 2-(N-Methyl-perfluorooctane sulfonamido) acetic acid; PFDA, perfluorodecanoic acid; EtFOSAA, 2-(N-Ethyl-perfluorooctane sulfonamido) acetic acid. PFAS were log-2 transformed, except for PFDA and EtFOSAA, which were analyzed as categorical variables.

Adjusted models were adjusted for parity, prenatal vitamin intake, maternal race/ethnicity, household income, maternal age, log 2 maternal cotinine, and an interaction term for the PFAS of interest and prenatal vitamin intake.

A p-interaction <0.10 was considered statistically significant.

Supplemental Table 7. Studies on per- and polyfluoroalkyl substances (PFAS) and folate levels

Reference	Population (age)	Year of Assessment	Sample Size	PFAS Assessed and matrix	Folate Assessed	Covariates	Primary Findings
Jain R (2021)[1]	NHANES (≥ 20 years)	2007-2014 [Cross-sectional]	N=6,291	▪ PFHxS<br>▪ PFOS<br>▪ PFOA<br>▪ PFNA<br>▪ PFDA<br>▪ PFUnDA<br><br>Matrix:<br>Blood serum	▪ RBC folate	▪ Age<br>▪ Gender<br>▪ Race/ethnicity<br>▪ Poverty: income ratio<br>▪ Smoking status (smoker: non-smoker)<br>▪ Survey years<br>▪ Last 24-hour consumption of folates, caffeine, and alcohol<br>▪ Last 30-day consumption of folates from dietary supplements	▪ RBC folate unweighted geometric mean in the total population was 1102 nmol/L (95% CI : 1077-1127)<br><br>▪ For PFOA, PFOS, PFDA, PFHxS, and PFNA, a 10% increase in concentration corresponded with a decrease in RBC folate of 0.33%, 0.66%, 0.83%, 0.16%, and 0.89%, respectively<br><br>Secondary Analyses<br>▪ No clear differences were observed by age<br>▪ Significant associations for PFOA and PFHxS were seen in females but not males.<br>▪ Those who were White not-Hispanic or other race had significant associations for PFHxS where those who were not-Hispanic black or Hispanic did not.

Tian Y (2022)[2]	NHANES<br><br>(adolescents)	2007-2010<br><br>[Cross-sectional]	N= 721	▪ PFHxS<br>▪ PFOS<br>▪ PFOA<br>▪ PFNA<br>▪ PFDA<br><br>▪ Mixture of five PFAS (PFHxS, PFOS, PFOA, PFNA, PFDA)<br><br>Matrix:<br><br>Blood serum	▪ Serum folate<br><br>▪ RBC folate	▪ Age<br>▪ Race/ethnicity<br>▪ Poverty: income ratio<br>▪ folate supplementation<br>▪ whole blood hemoglobin<br>▪ serum cotinine concentrations<br>▪ survey year	▪ Mean serum folate concentrations: 41.2 nmol/L<br>▪ Mean RBC folate concentrations: 947.0 nmol/L<br><br>▪ Unit increase in serum PFOS, PFOA, PFNA, and PFDA associated with decrease in RBC folate concentrations (nmol/L) of 72.4 (-112.7, -32.2), 58.3 (-115.0, -1.6), 60.7 (-107.5, -13.8), and 76.5 (-119.0, -33.9), respectively<br>▪ Inverse associations between serum folate and serum PFDA, with a unit increase in serum PFDA associated with a serum folate (nmol/L) decrease of 2.4 (-4.7, -0.1)<br><br>▪ PFAS mixture inversely associated with RBC folate (BKMR and QGcomp) with ß = -47.9 (-78.6, -17.1) for QGcomp model<br><br><u>Secondary Analyses</u><br><br>▪ Modification by age was found for serum folate and PFHxS and PFOA (inverse associations)<br>▪ No modification by sex or race/ethnicity were observed
Zhang Y (2023)[3]	NHANES<br><br>(adolescents and adults)	2003-2016<br>[Cross-sectional]	N=11,961	▪ PFHxS<br>▪ PFOS<br>▪ PFOA<br>▪ PFNA	▪ Serum folate<br><br>▪ RBC folate	▪ Age<br>▪ Sex<br>▪ Race and ethnicity	<u>Adolescents</u><br>▪ RBC folate geometric mean: 353.4 ng/mL<br>▪ Serum folate geometric mean: 15.4 ng/mL

	(2,802 adolescents and N=9,159 adults)	Matrix:<br><br>Blood serum	▪ BMI<br>▪ Poverty-to-income ratio<br>▪ Survey cycle<br>▪ Healthy eating index 2010	▪ 2.7-fold increase in RBC folate associated with reductions in PFOS and PFNA concentrations of 24.36% (-33.21, -14.34) and 13.00% (-21.87, -3.12)<br>▪ 2.7-fold increase in serum folate associated with an 11.57% (-17.87, -4.79) decrease in PFOS concentrations<br><br><u>Adults</u><br><br>▪ RBC folate geometric mean: 408.1 ng/mL<br>▪ Serum folate geometric mean: 14.9 ng/mL<br>▪ 2.7-fold increase in RBC folate associated with reductions in PFOA, PFOS, PFNA, and PFHxS concentrations of 12.45% (-17.28, -7.35), 25.3% (-29.67, -20.65), 21.65% (-26.19, -16.82), and 11.7% (-17.32, -5.7), respectively<br>▪ 2.7-fold increase in serum folate associated with reductions in PFOA, PFOS, and PFNA concentrations of 5.01% (-8.35, -1.54), 12.81% (-16.15, -9.35), and 11.85% (-14.72, -8.88), respectively

References

1. Jain, R. B. (2021). Impact of the increasing concentrations of selected perfluoroalkyl acids on the observed concentrations of red blood cell folate among US adults aged ≥20 years. Environmental Science and Pollution Research, 28(37), 52357-52369. https://doi.org/10.1007/s11356-021-14454-9

2. Tian, Y., Luan, M., Zhang, J., Yang, H., Wang, Y., & Chen, H. (2022). Associations of single and multiple perfluoroalkyl substances exposure with folate among adolescents in NHANES 2007–2010. Chemosphere, 307, 135995. https://doi.org/10.1016/j.chemosphere.2022.135995

3. Zhang, Y., Mustieles, V., Wang, Y.-X., Sun, Y., Agudelo, J., Bibi, Z., Torres, N., Oulhote, Y., Slitt, A., & Messerlian, C. (2023). Folate concentrations and serum perfluoroalkyl and polyfluoroalkyl substance concentrations in adolescents and adults in the USA (National health and nutrition examination study 2003–16): An observational study. The Lancet Planetary Health, 7(6), e449-e458. https://doi.org/10.1016/s2542-5196(23)00088-8

Supplemental Materials

Supplemental Information for *Associations of prenatal per- and polyfluoroalkyl substances with whole blood folate levels in pregnant women in the Health Outcomes and Measures of the Environment (HOME) Study*

Harin Lee*, Amber M. Hall*, Antonia M. Calafat, Aimin Chen, Zia Fazili, Bruce P. Lanphear, Christine M. Pfeiffer, Kimberly Yolton, and Joseph M Braun

*Dual first authors

Corresponding author:
Amber M Hall
Department of Epidemiology
Brown University
Providence, Rhode Island 02912, United States
email: amber_hall@brown.edu

Supplemental Figure 1. Study flow chart using data from the HOME Study (2003-2006).

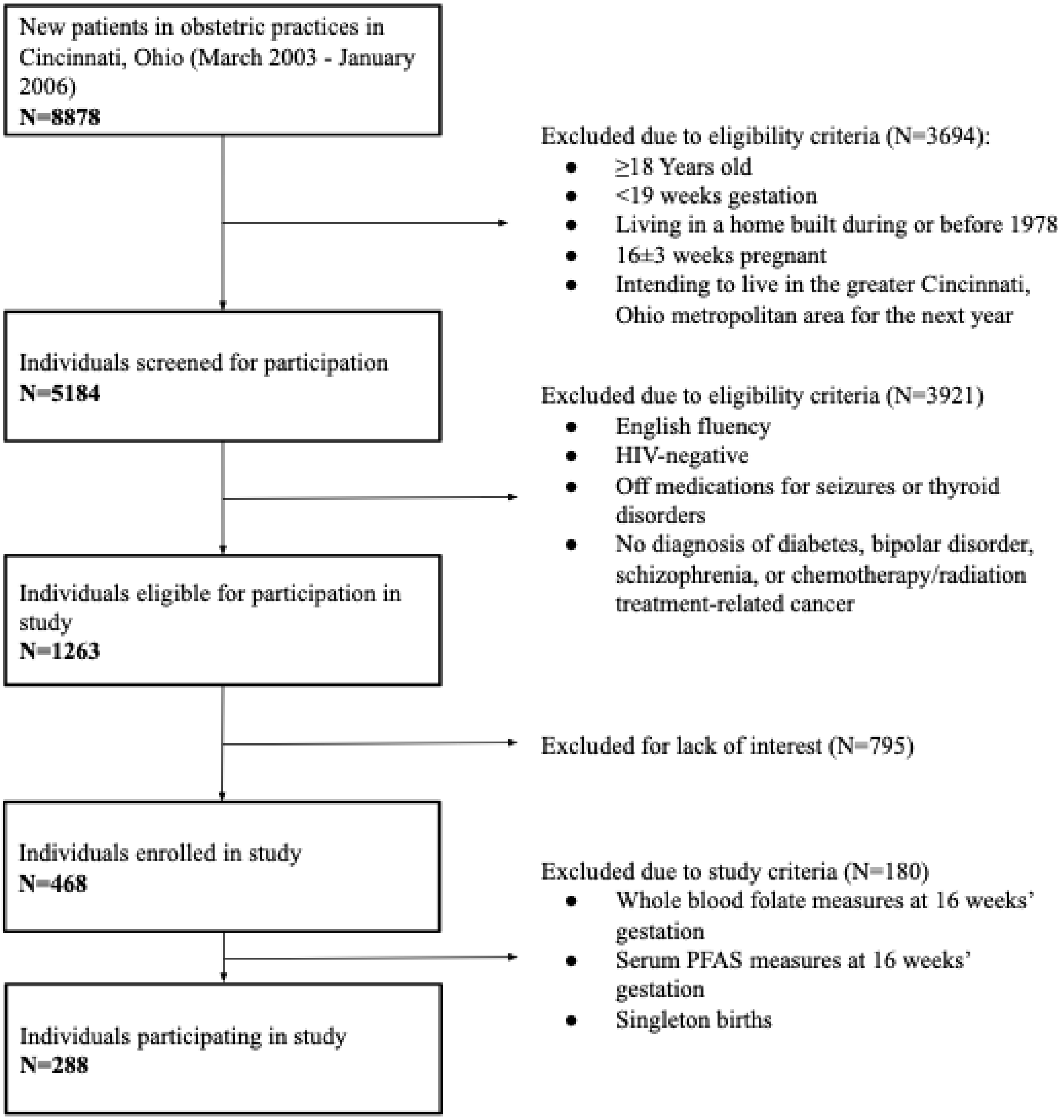

Note: HOME, Health Outcomes and Measures of the Environment.

Supplemental Figure 2. DAG of the relationship between PFAS and WBF in pregnant women (16 weeks' gestation)

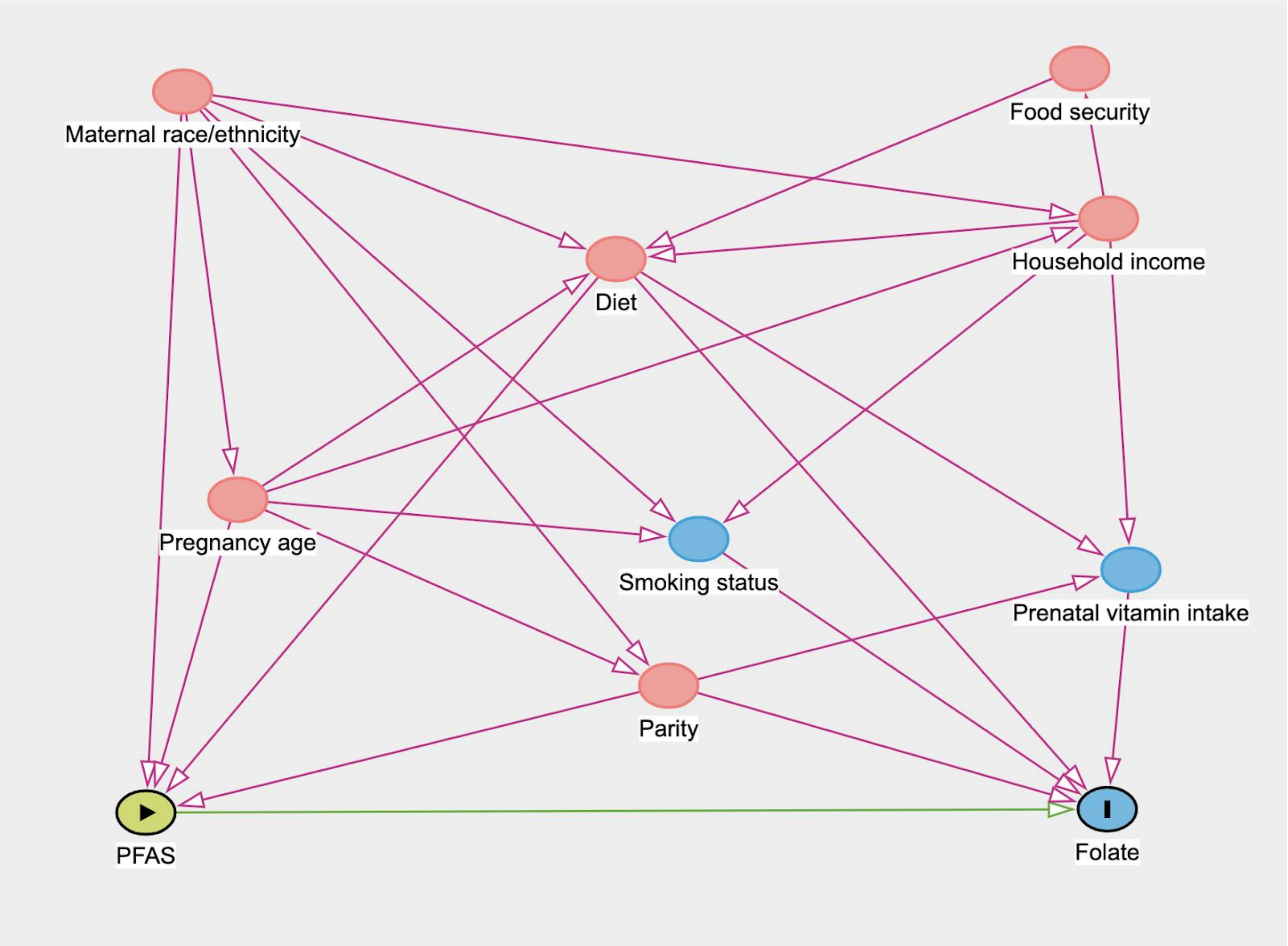

Note: DAG, Directed Acyclic Graph; PFAS, per- and polyfluoroalkyl substances; WBF, whole blood folate.

Bolded green is the exposure; bolded blue is the outcome; blue is an ancestor of the outcome; pink is a confounder.

Supplemental Figure 3. Pearson correlations between log2-transformed serum concentrations of PFAS in pregnant women at 16 weeks' gestation in the HOME Study (2003-2006), N=288

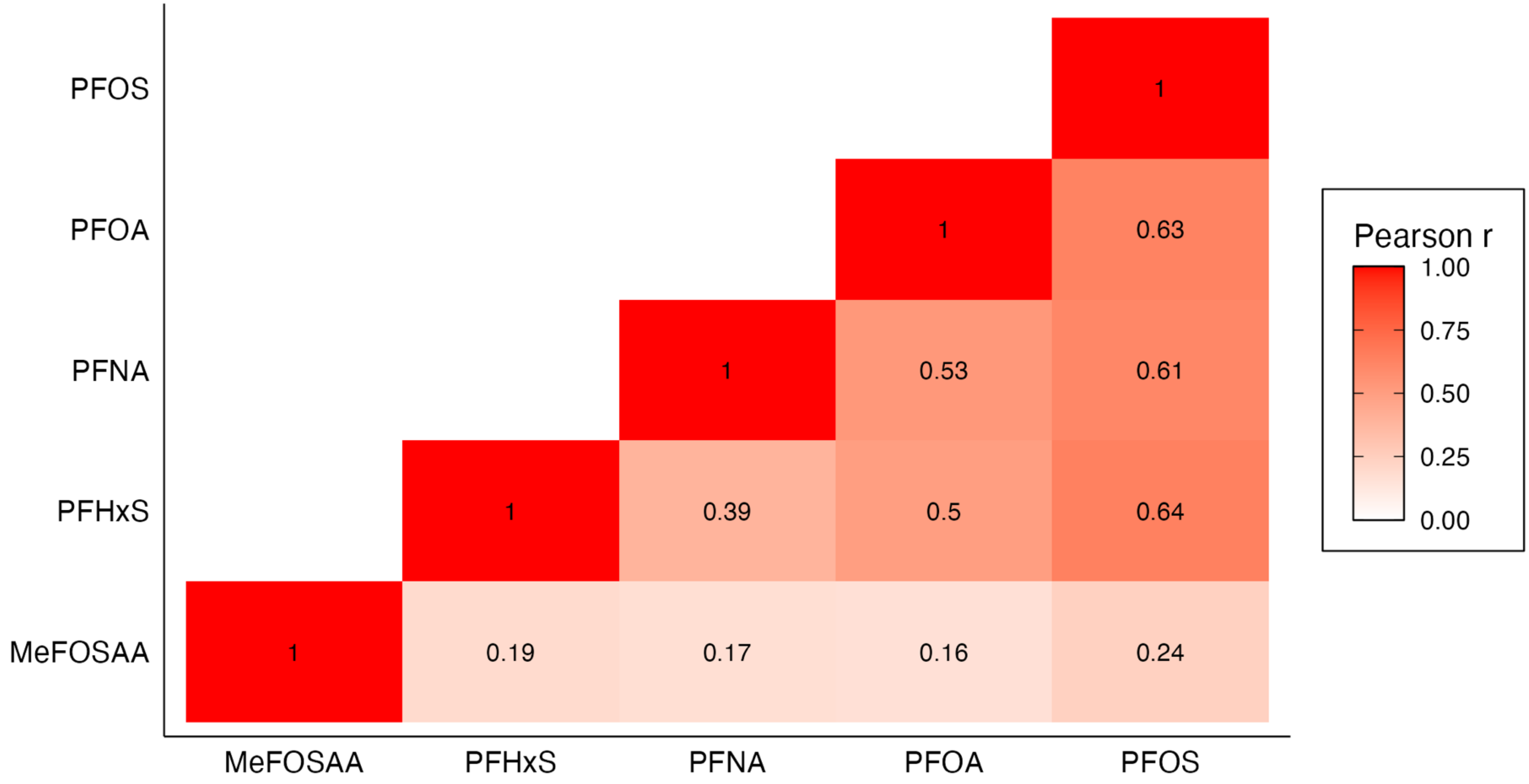

Note: PFAS, per- and polyfluoroalkyl substances; HOME, Health Outcomes and Measures of the Environment Study; PFOS, perfluorooctane sulfonic acid; PFOA, perfluorooctanoic acid; PFNA, perfluorononanoic acid; PFHxS, perfluorohexane sulfonic acid; MeFOSAA, 2-(N-Methyl-perfluorooctane sulfonamido) acetic acid.

One PFHxS concentration <limit of detection (LOD) was imputed as LOD/√2; concentrations for the other PFAS all detected in 100% of samples.

Supplemental Figure 4. Adjusted quantile-based g-computation model: negative and positive weights for PFAS mixture in pregnant women (16 weeks' gestation), HOME Study (2003-2006), N=288

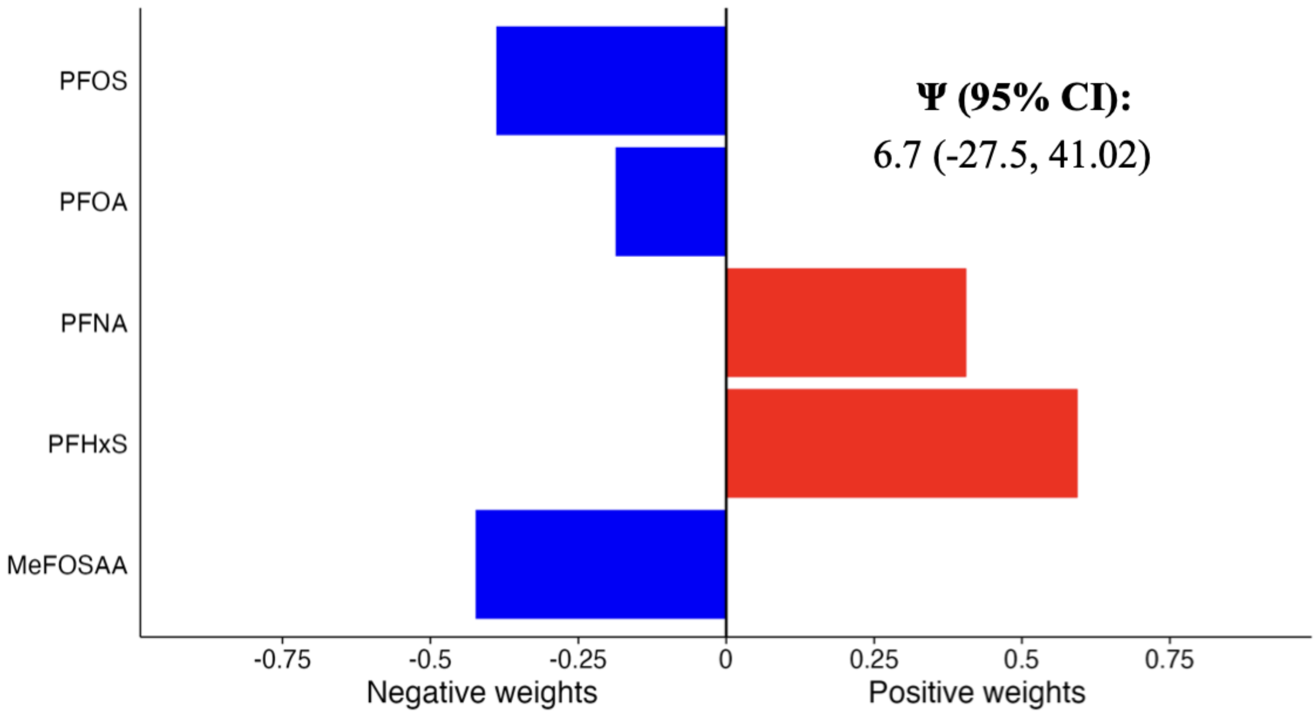

Note: PFAS, per- and polyfluoroalkyl substances; HOME, Health Outcomes and Measures of the Environment Study; PFOS, perfluorooctane sulfonic acid; PFOA, perfluorooctanoic acid; PFNA, perfluorononanoic acid; PFHxS, perfluorohexane sulfonic acid; and MeFOSAA, 2-(N-Methyl-perfluorooctane sulfonamido) acetic acid.

This figure shows the fixed negative and positive mixture weights from a quantile-based g-computation model evaluating joint PFAS effects for the PFAS (PFOS, PFOA, PFNA, PFHxS, MeFOSAA) on total folate concentrations, adjusting for parity, prenatal vitamin intake, maternal race/ethnicity, household income, maternal age, and log 2 maternal cotinine.

Supplemental Figure 5. Dose-response curves for the relationship between WBF and individual PFAS z-scores in a BKMR model when all other PFAS are set at the median in the HOME Study (2003-2006), N=288

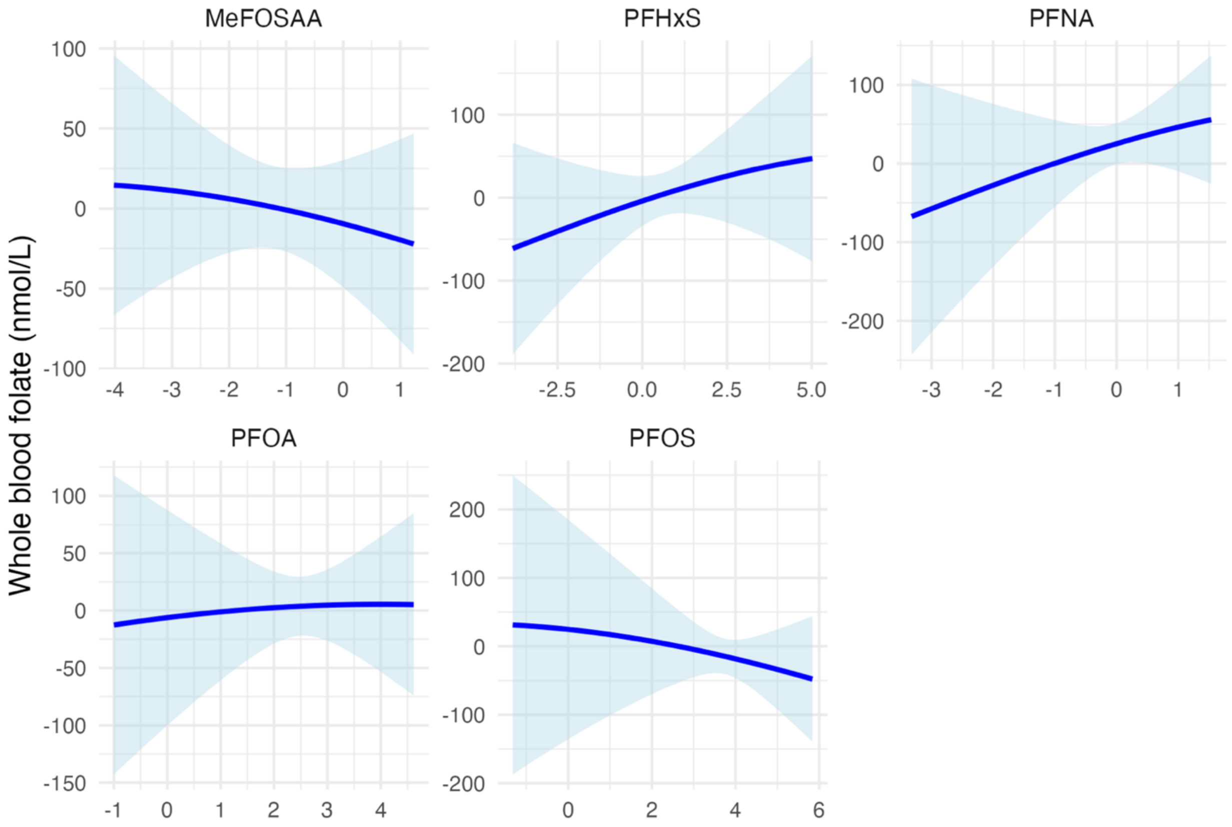

Note. WBF, whole blood folate; PFAS, per- and polyfluoroalkyl substances; BKMR, Bayesian Kernel Machine Regression; HOME, Health Outcomes and Measures of the Environment; MeFOSAA, 2-(N-Methyl-perfluorooctane sulfonamido) acetic acid; PFHxS, perfluorohexane sulfonic acid; PFNA, perfluorononanoic acid; PFOA, perfluorooctanoic acid; PFOS, perfluorooctane sulfonic acid; nmol/L, nanomoles per liter.

Shading indicates 95% confidence intervals. PFAS were measured using ng/mL and log-2 transformed. The model used a Gaussian kernel, Markov Chain Monte Carlo algorithm, and ran 50,000 iterations. Models were adjusted for parity, prenatal vitamin intake, maternal race/ethnicity, household income, maternal age, and log 2 maternal cotinine
